# Immune challenge reduces gut microbial diversity and triggers fertility-dependent gene expression changes in a social insect

**DOI:** 10.1186/s12864-020-07191-9

**Published:** 2020-11-23

**Authors:** Matteo Antoine Negroni, Francisca H. I. D. Segers, Fanny Vogelweith, Susanne Foitzik

**Affiliations:** 1 Institute of Molecular and Organismic Evolution, Johannes Gutenberg University, Hanns-Dieter-Hüsch-Weg 15, 55128 Mainz, Germany; 2 Department for Applied Bioinformatics, Inst. of Cell Biology and Neuroscience, Goethe University, Frankfurt, Germany; 3 LOEWE Centre for Translational Biodiversity Genomics (LOEWE-TBG), Frankfurt, Germany; 4 M2i Biocontrol, Parnac, France

**Keywords:** Immunity, Gut microbiome, Fecundity, Aging, Senescence, Longevity, Social insects

## Abstract

**Background:**

The gut microbiome can influence life history traits associated with host fitness such as fecundity and longevity. In most organisms, these two life history traits are traded-off, while they are positively linked in social insects. In ants, highly fecund queens can live for decades, while their non-reproducing workers exhibit much shorter lifespans. Yet, when fertility is induced in workers by death or removal of the queen, worker lifespan can increase. It is unclear how this positive link between fecundity and longevity is achieved and what role the gut microbiome and the immune system play in this. To gain insights into the molecular regulation of lifespan in social insects, we investigated fat body gene expression and gut microbiome composition in workers of the ant *Temnothorax rugatulus* in response to an experimental induction of fertility and an immune challenge.

**Results:**

Fertile workers upregulated several molecular repair mechanisms, which could explain their extended lifespan. The immune challenge altered the expression of several thousand genes in the fat body, including many immune genes, and, interestingly, this transcriptomic response depended on worker fertility. For example, only fertile, immune-challenged workers upregulated genes involved in the synthesis of *alpha-ketoglutarate*, an immune system regulator, which extends the lifespan in *Caenorhabditis elegans* by down-regulating the TOR pathway and reducing oxidant production. Additionally, we observed a dramatic loss in bacterial diversity in the guts of the ants within a day of the immune challenge. Yet, bacterial density did not change, so that the gut microbiomes of many immune challenged workers consisted of only a single or a few bacterial strains. Moreover, the expression of immune genes was linked to the gut microbiome composition, suggesting that the ant host can regulate the microbiome in its gut.

**Conclusions:**

Immune system flare-ups can have negative consequence on gut microbiome diversity, pointing to a previously underrated cost of immunity. Moreover, our results provide important insights into shifts in the molecular regulation of fertility and longevity associated with insect sociality.

## Background

Why and how organisms age are long-standing scientific questions [1–4]. Senescence is often considered a consequence of the progressive accumulation of molecular damage due to incomplete somatic repair [5], which results in a decline in physiological function including immunity and homeostasis [6]. Endogenous causes of molecular damage are diverse, and include the production of reactive oxygen species (ROS) through metabolic activity, shortening of telomeres and spontaneous errors in replication, transcription and translation [6–10]. Hence, in an environment free of extrinsic risks (e.g., starvation, predation, pathogens), lifespan ultimately depends on two antagonist processes that determine the ability to maintain the soma: the rate of molecular damage and the efficiency at which such molecular damage is repaired [4, 6, 11]. The well-conserved *insulin/insulin-like* growth factor (IIS) and the *target of rapamycin* (TOR) signalling pathways are important regulators of longevity that underlie the negative association between somatic maintenance and reproduction observed in most organisms [12, 13]. While some molecular processes underlying lifespan are well understood, the importance of other factors may have been underestimated. For instance, the influence of diet on lifespan via the ISS and TOR pathways is well studied [14], whereas less attention has been paid to the gut microbiome [15] and the immune system. Yet, recent studies suggest that both play an important role. In *Drosophila,* host fitness is tightly linked to the microbiome and fly guts promote a high microbial diversity. Interactions between gut bacteria affect both fecundity and longevity of *Drosophila* flies, but in opposite directions [16, 17]. Also in social bees the composition of the gut microbiome community results from a balance between the immune system of the host and bacterial proliferation [18]. The role of immunity alone or in combination with microbial partners are likely important, by rarely considered regulators of lifespan and fecundity [19, 20].

In most organisms, longevity is traded-off with reproduction, but in social insects, these two life-history traits are positively associated [21]: the most fertile individuals in insect societies, the queens, live the longest [22, 23]. Longevity is a phenotypic plastic trait, as the extreme variation in lifespan between the reproductive queen and the non-reproductive worker castes likely arise from the same genetic background [24]. The positive association between fecundity and longevity does not only hold when queens and workers are compared, but is also observed within a single caste. Indeed, differences in lifespan can not only be set during development, but also during the adult life of a social insect. For example, in *Temnothorax* ants, despite the fact that workers cannot mate, queen removal induces ovary development and the laying of haploid, male-destined eggs in workers, which extends their lifespan by 13% [23]. Moreover, this impact of queen absence on fecundity and survival does not only affect the few fertile workers in the colony, but to some degree all workers, as dominant workers are less able to inhibit ovary development in their nestmates [23, 25]. What remains unclear is, how this lifespan extension, as well as the general positive link between fecundity and longevity, is achieved in social insects on a molecular and physiological level. Social insects provide thus a unique opportunity to investigate intrinsic and extrinsic regulators of lifespan [26].

Here we propose three non-mutually exclusive hypotheses to explain the increase in worker lifespan with fertility in response to queen removal. First, the lifespan extension of fertile workers could be explained by a higher investment into body maintenance. Possibly, fertile workers extend their lifespan by investing more energy into i) a reduction of ROS production, ii) a better resistance to oxidative stress (e.g., via production of antioxidants) [27, 28], and/or iii) better repair mechanisms. The uncoupling or the positive link between longevity and fecundity in social insects suggests a reshaping of those pathways and/or modifications in their regulation compared to solitary organisms [26].

Second, the increase in worker longevity could stem from changes in the microbial community of the gut [29]. IIS and TOR signalling pathways are nutrient sensitive, thus the gut bacteria could affect the regulation of longevity pathways via the digestion and absorption of specific nutrients by the host [30]. In line with this hypothesis, the microbiome diversity changes with biological age, and in *Drosophila* gut microbiome composition has been causally linked to aging and lifespan [17, 31]. Moreover, gut bacteria can also be beneficial for immune defence. In *Caenorhabditis elegans*, inoculation through feeding on non-pathogenic bacteria *Lactobacilli* and *Bifidobacteria* not only extends lifespan but also improves pathogen resistance [32, 33]. The positive effect of gut bacteria on growth and pathogen resistance in honey bees is related to improved digestion and absorption of nutrients as well as to higher production of antimicrobial substances [30].

Finally, fertility could be linked to a stimulation of the immune system, so that increased pathogen resistance could extend lifespan. In *Lasius niger* mating activate the immune system of the queen, but in *Temnothorax* ants, workers that become fertile lay haploid egg without mating. Immunity is typically costly for body maintenance and impairs survival [34]. For instance, a non-lethal immune challenge reduces lifespan in bumblebee workers [20], likely through the production and activity of radical oxygen species (ROS). If the lifespan extension in ant workers would involve molecular changes in immunity, fertile workers should alter expression of immune genes, an effect that might be more detectable when the immune system is provoked.

**Fig. 1:**
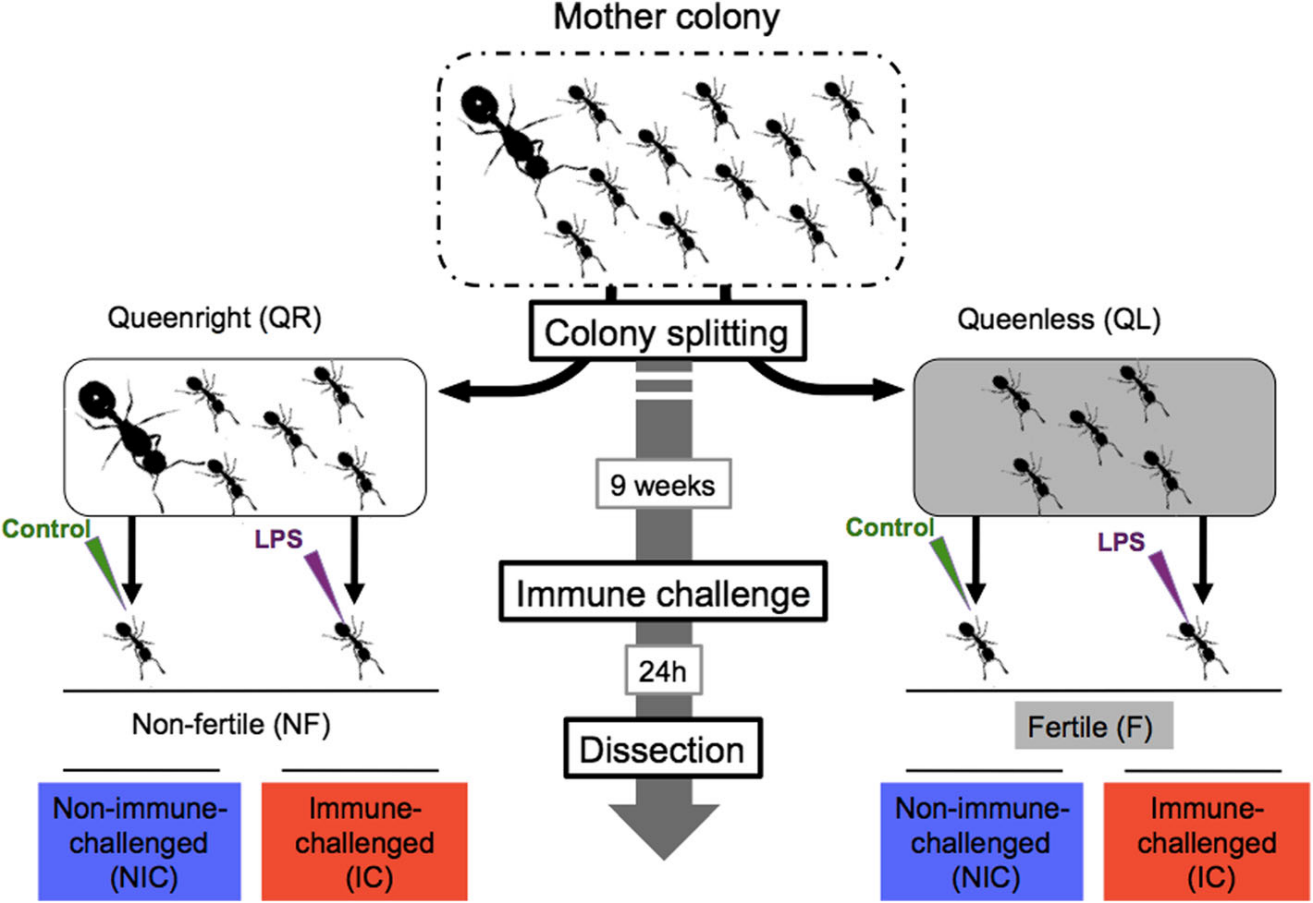
Experimental design. Eight monogynous mother colonies were equally split in two fragments: one containing the queen (queenright: QR) and the other being queenless (QL). After 9 weeks, two workers were sampled from each nest. One was immune-challenged (IC) by pricking with a needle dropped into a 10% lipo-polysaccharide solution (LPS-PGN), while the other non-immune-challenged worker (NIC) received the same manipulation, but the needle only touched the cuticle. Twenty-four hours after the manipulation, the fat body was dissected for RNA extraction, the gut for 16S microbiome sequencing and the ovaries for size measurements. QL workers had longer ovaries than workers from the QR nests (fertile workers F; non-fertile workers NF). The figure was designed by Matteo Negroni using photos of *T. rugatulus* taken by M. Negroni and S. Foitzik

In this study, we investigated these three hypotheses by studying the molecular regulation of lifespan, and it’s link to immunity and gut microbiome composition in workers of the ant *Temnothorax rugatulus* [35]. First, we showed in a long-term survival experiment that worker fertility induced by queen removal extends worker lifespan also in our focal species. Then we subjected these fertile and non-fertile workers to a non-lethal immune challenge and compared gut bacterial diversity as well as their transcriptomic responses in the fat body (Fig. 1), the primary organ of systemic immunity in insects [36]. If fertile workers live longer because of an increased investment in body maintenance, we would predict an upregulation of genes involved in molecular repair mechanisms or antioxidant production. If changes in microbial composition of the gut are contributing to the increased longevity of fertile ant workers, we would expect an impact of worker fertility on the composition of the gut microbiome. Finally, the extension in worker lifespan may be explained by an improved immuno-competence. If so, we envisaged that i) the baseline expression of immune genes differs between fertile and non-fertile workers and ii) the immune response following an immune challenge depends on worker fertility. We show below that fertile workers invest more into repair mechanisms and other somatic maintenance functions, especially when their immune system is challenged. This might explain their lifespan extension, particularly when exposed to pathogens. The microbial composition of the gut is not involved in the lifespan shifts following fertility induction. However, the immune challenge had a severe impact on the gut microbiome by slashing microbial diversity, and these changes in the composition of the gut microbiome were linked to the expression of important immune genes.

## Results

### Influence of queen removal on worker survival and fertility

**Fig. 2:**
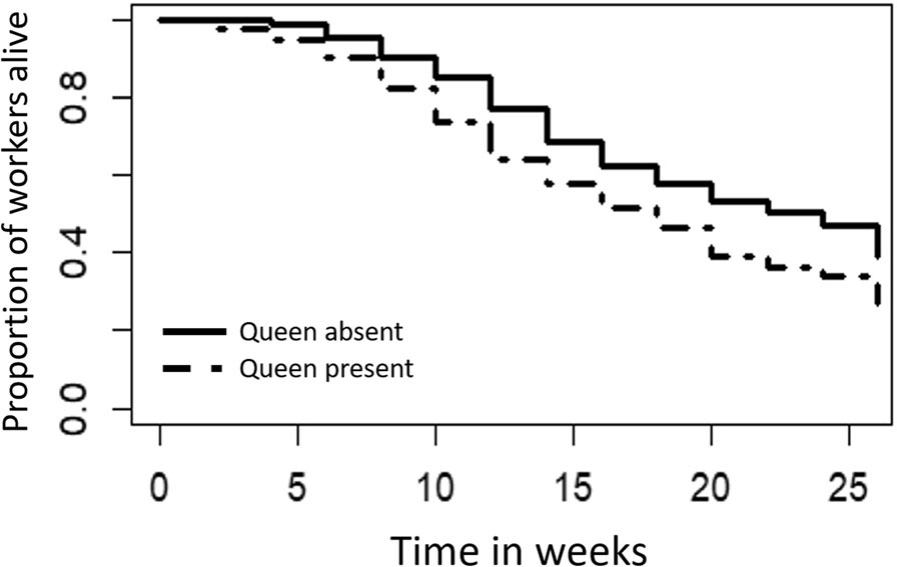
Worker survival over time under the presence of the queen (dashed lines) or her absence (solid line). Survival analysis revealed a higher survival in the absence than in the presence of the queen (Cox survival mixed-effects model; x^2^ = 18.9; df = 1; *P* < 0.0001)

Queen removal led to an increase in worker survival by 12.5% in queenless colony fragments over 26 weeks (cox survival mixed-effects model: X^2^ = 18.9; df = 1; *P* < 0.0001; Fig. 2). Furthermore, *T. rugatulus* workers in queenless colony fragments showed a 28.6% growth in ovary length within 9 weeks (mean ovariole length ± SD; queenright: 0.49 ± 0.14 mm; queenless: 0.63 ± 0.19 mm; F_1_ = 5.55, *p* = 0.027).

### Influence of fertility and immune challenge on fat body gene expression

We conducted a full-factorial experimental design manipulating the immune system and worker fertility. We challenged the immune system of workers from queenright and queenless experimental colonies by pricking them between the 2nd and 3rd gaster segment with a needle dipped in a 10% lipo-polysaccharide solution. Control workers received the same manipulation, but the needle only touched the cuticle, not penetrating it. After 24 h, we removed the fat body and gut of the focal workers (*N* = 32) to analyse gene expression via RNAseq and the gut microbiome community by 16S sequencing.

**Fig. 3:**
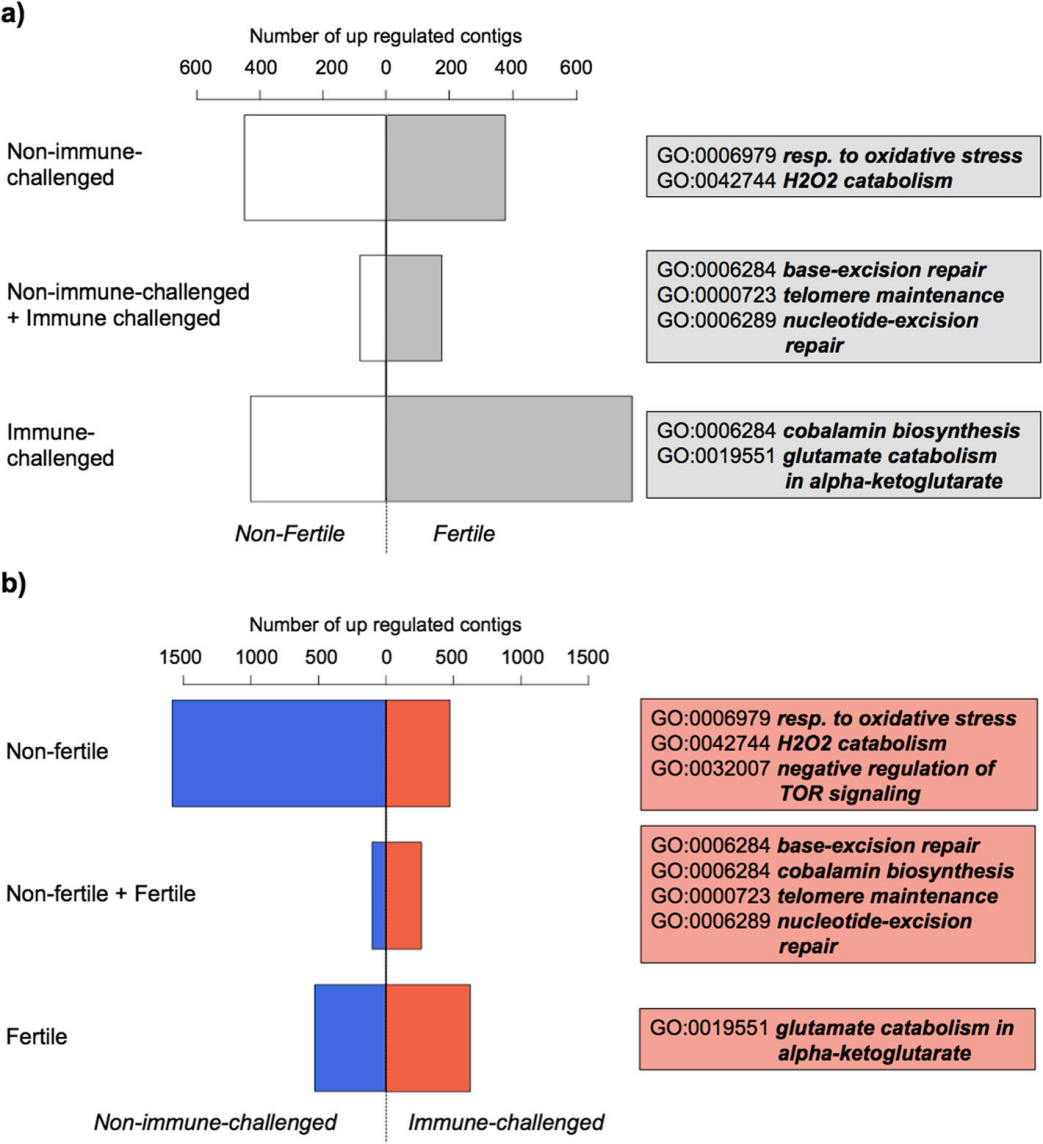
Number of differentially expressed genes and their functional enrichments influenced by the interaction between the immune challenge and the fertility induction: **a** comparison of upregulated genes in fertile (grey bars) and infertile (white bars) workers within the non-challenged and the immune-challenged treatment groups and irrespective of the immune challenge (*non-immune-challenged* + *immune-challenged*) **b**) comparison of upregulated genes in non-challenged (blue bars) and immune-challenged (red bars) workers, within the non-fertile and fertile treatment groups as well as irrespective from the fertility induction

Fertility induction and the immune system provocation interactively altered the expression of 2387 genes (Table S8). More than (55%) half of them displayed opposing expression patterns in response to the queen’s presence and immune challenge (Fig. S1). We consequently compared i) fertile vs. non-fertile workers, separately for the immune and non-immune-challenged workers, and ii) immune-challenged vs. non-challenged workers, separately for fertile and infertile workers [37]. Fertility induction was associated with differential expression of 1466 genes in the immune-challenged workers and 1101 genes in the non-challenged workers (Fig. 3a; Table S9). Conversely, the immune challenge resulted in an altered expression of 2422 genes in non-fertile workers, whereas 1518 genes changed their expression in fertile workers (Fig. 3b; Table S10).

Both fertility induction and immune challenge led to a changed expression of genes involved in various molecular repair mechanisms (Fig. 3b; Table S12). Fertile workers activated genes of the *glutamate degradation into alpha-ketoglutarate* pathway in response to the immune challenge (Fig. 3a, b, Table S11, S12). Non-immune-challenged workers stimulated processes involved in oxidative stress reduction (*response to oxidative stress, H*_*2*_*O*_*2*_ *catabolism*) in response to the fertility induction (Fig. 3a, Table S11), whereas infertile workers upregulated these functions following the immune challenge (Fig. 3b, Table S12).

**Fig. 4:**
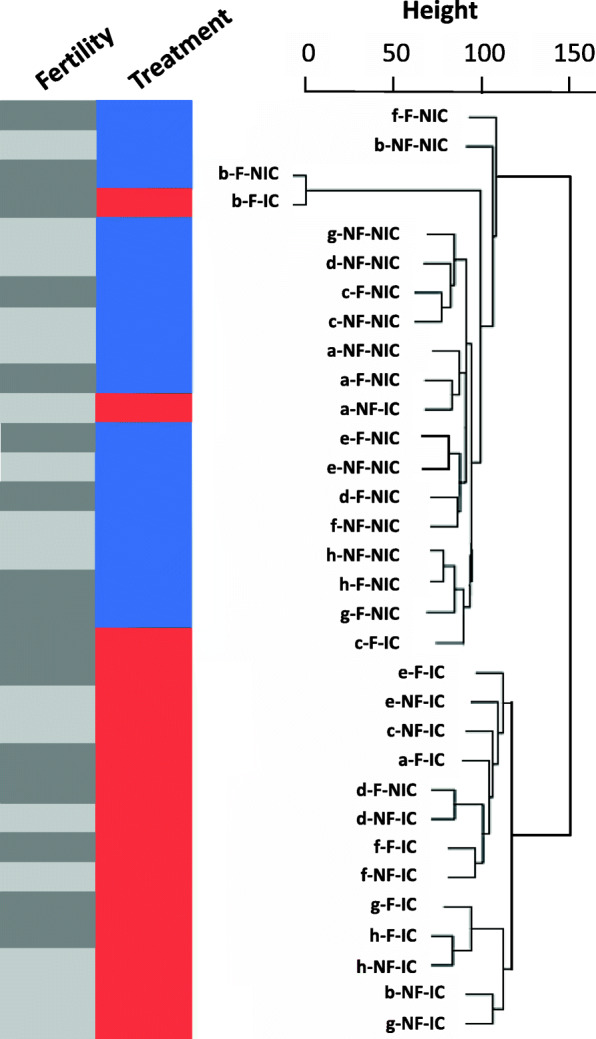
Dendrogram showing clustering of samples according to fertility (non-fertile: NF and green; or fertile: F and red), and treatment (non-immune-challenged: NIC and light grey; or immune-challenged: IC and dark grey)

Transcriptomes clustered mainly by whether or not the immune system of the workers was challenged (Fig. 4), which was reflected in the main effects of the expression analysis. Fertility induction only changed the expression of 4268 genes independently of the immune challenge. Three thousand one hundred twenty-nine genes were upregulated in infertile workers and 1139 in fertile ones, among the latter the *vitellogenin receptor* (complete list, Table S13). Fertile workers upregulated genes with the function *S-adenosylmethionin biosynthetic process* (Table S14a, b) This process is not only important for protein synthesis, but also plays a role in the molecular regulation of longevity, as a knockdown of *S-adenosylmethionin synthase* extends lifespan in *Caenorhabditis elegans* [38]*.*

**Fig. 5:**
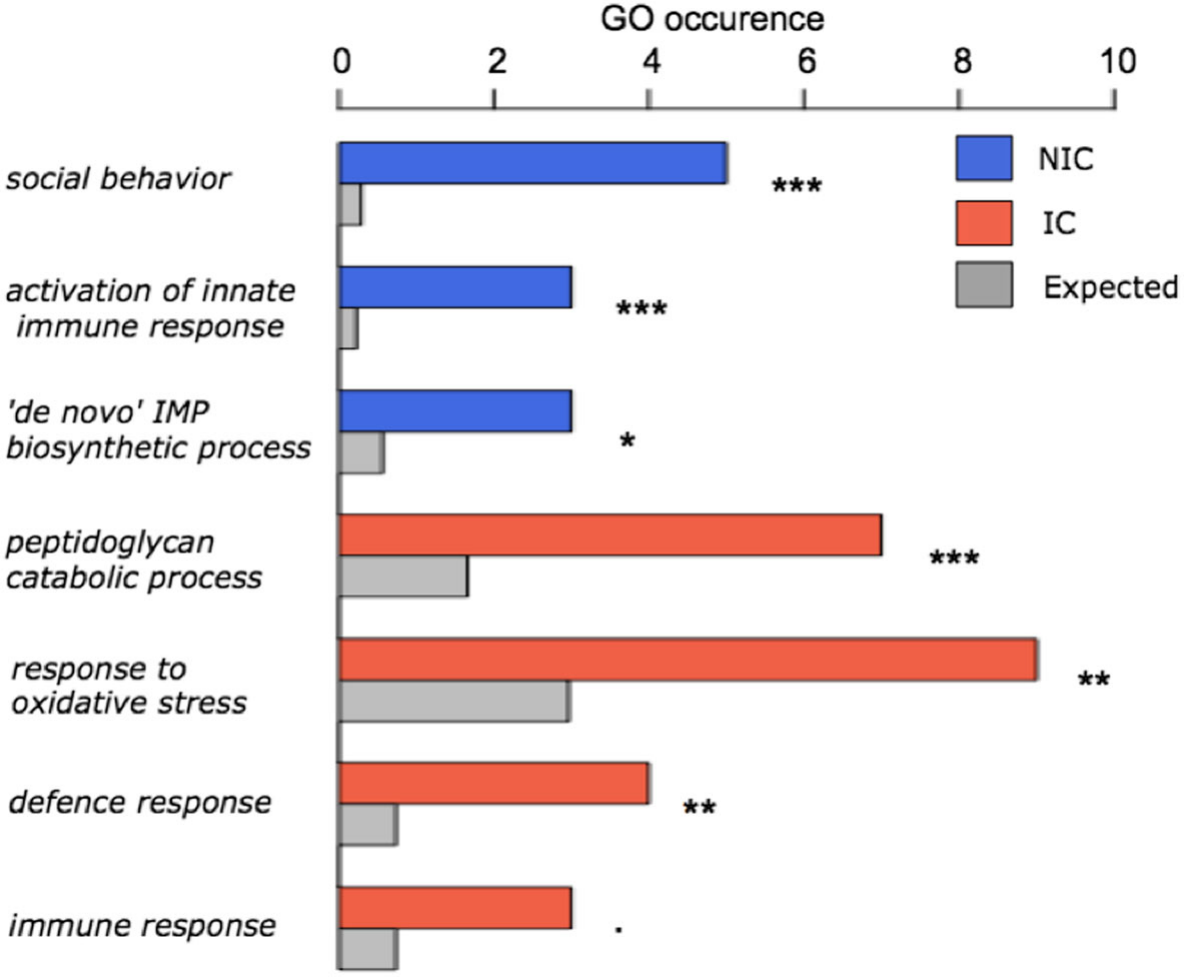
Gene ontology term (GO) representation of relevant functions from the functional enrichment analysis (significance code: *: < 0.05; **: < 0.01; ***: < 0.001; the dot represents marginal enrichment for a *p* = 0.056). The number of times a function occurred in the list of upregulated genes for the non-immune-challenged (NIC) workers, immune-challenged (IC) workers, and the expected number (following a random distribution) are coloured blue, red and grey, respectively

More than twice as many genes (10,717) altered their expression in response to the immune challenge (independently from fertility) than did the fertility induction following the removal of the queen (independently from the immune challenge). Six thousand one hundred thirty-six genes were upregulated in the immune-challenged workers, while 4581 were more highly expressed in non-immune-challenged workers (Table S15). The enrichment analysis of the overexpressed genes in the immune-challenged workers revealed functions related to immunity, such as *peptidoglycan catabolic process*, *defence response*, de novo *IMP biosynthetic process* and *innate immune response*, or stress reactions such as *response to oxidative stress* (Fig. 5, Table S14c, d). *Immune response* was only marginally enriched in immune-challenged workers (*P* = 0.056). Interestingly, non-immune-challenged workers upregulated genes with enriched functions such as *innate immune response* and *social behaviour*.

### Influence of fertility and immune challenge on the gut microbiome

**Fig. 6:**
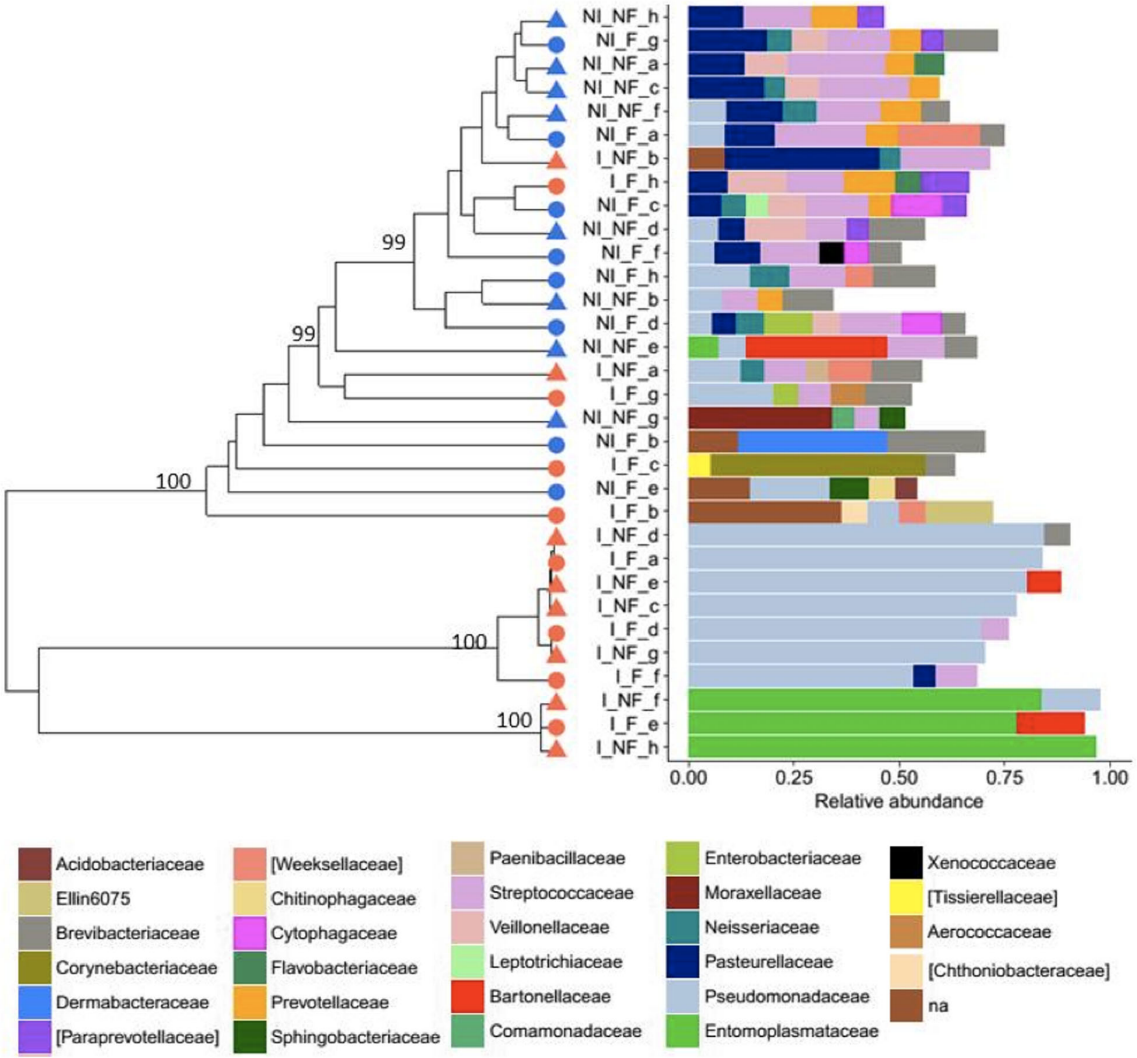
Clustering of samples based on the Bray-Curtis dissimilarities using the average linkage method. Clusters with AU values > 98.5 are highly supported clusters and are indicated on the tree with their respective AU values. Red and blue symbols at the tree tips represent the samples from immune-challenged and non-immune-challenged ants, respectively. Fertile and non-fertile workers are indicated by, respectively, circles and triangles. Samples that share the same letter (a to h) originate from ants belonging to the same colony. The relative abundances (≥0.05) of microbial families are plotted next to the samples. The OTUs which did not have a family assigned were binned in the category “na”

**Fig. 7:**
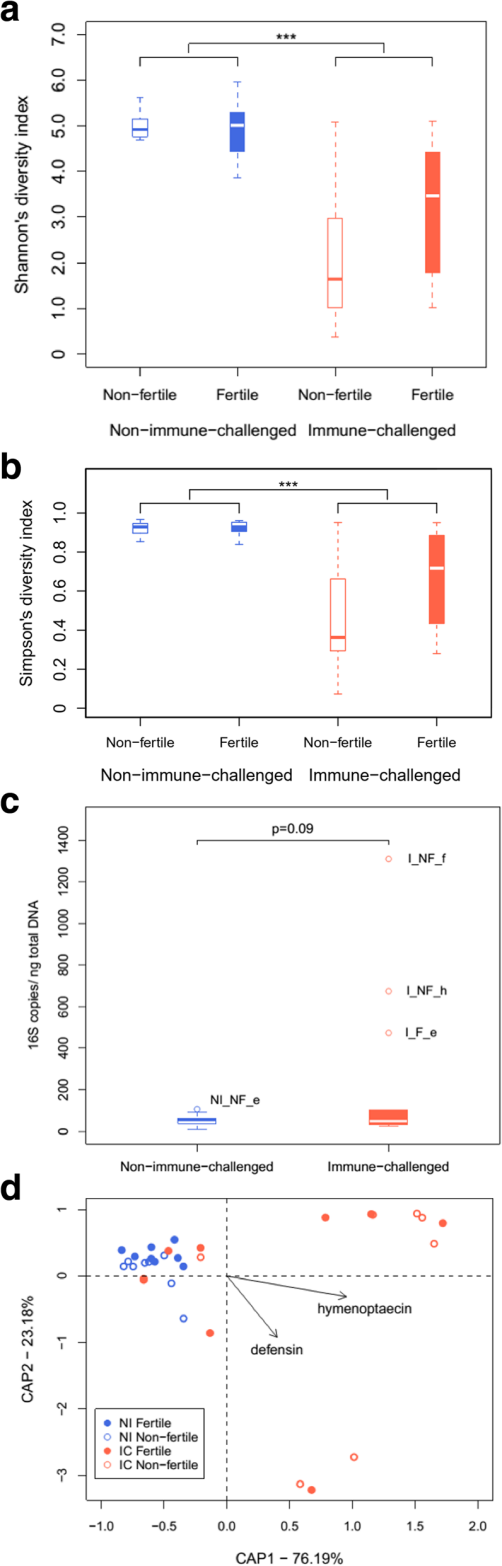
Diversity indexes grouped by treatment. The horizontal bars in the boxplots represent the medians and the boxes indicate the first and third quartile. Immune-challenged and non-immune-challenged ants harboured gut communities that differ significantly in **a**) Shannon’s diversity and **b**) Simpson diversity index as indicated by the asterisks on the plot. The gut communities of immune-challenged ants had a lower diversity than those of non-immune-challenged ants. **c**) The number of 16S rRNA copies per ng of total DNA grouped by immunity treatment Samples originating from immune-challenged ants tended to have more copies of the 16S rRNA gene. The samples outside the third quartile are represented by dots and their names, corresponding to those used in Fig. 6, are written on the plot. **d**) Constrained ordination biplot of the *T. rugatulus* gut communities showing the association between the expression of the antimicrobial effector genes *hymenoptaecin* and *defensin* (TPM) with the bacterial community variance. The length of the arrows reflects the strength of the correlation of each gene with the gut communities

The analysis of pairwise Bray-Curtis dissimilarities between samples revealed no clustering of gut communities based on worker fertility (Fig. 6). Moreover, both the Shannon’s diversity and Simpson’s diversity index did not differ between the gut communities of fertile and infertile ants (Linear mixed-effects model, Fertility: F = 1.75, *P* = 0.23; Fertility × Immune challenge: F = 1.72, *P* = 0.21 and Fertility: F = 2.20, *P* = 0.18; Fertility × Immune challenge: F = 1.82, *P* = 0.20, respectively). In contrast, most of the gut communities from immune-challenged ants clustered separately from the ants that did not undergo this treatment (Fig. 6). In agreement with these results, the constrained ordination analysis indicated that only the immune challenge and not the fertility induction explained gut community variance (Immune challenge: F = 4.64, *P* = 0.01; Fertility: F = 0.72, *P* = 0.71; Fertility × Immune challenge: F = 0.78, *P* = 0.63). The immune challenge greatly reduced diversity in the microbial communities in the gut as evidence by both the Shannon’s and Simpson’s diversity indices (Linear mixed-effects models, F = 30.12, *P* < 0.001 and F = 28.18, *P* < 0.001, respectively; Fig. 7a and b).Three immune-challenged ants showed gut communities composed mainly of a single OTU assigned to the bacterial family Entomoplasmataceae and the intracellular genus *Entomoplasma*, while the gut communities of seven ants were dominated by Pseudomonaceae, mainly belonging to a single OTU from the genus *Pseudomonas* (Fig. 6).

Worker fertility did not affect the relative abundance of microbial families (Table S16), suggesting that the fertility induction did not alter the community composition of the gut. In contrast, 20 of the 64 examined bacterial families contributed less to the gut communities of immune-challenged workers compared to the non-immune-challenged ones (Table S16). The two families that made up the largest parts of the gut community of immune-challenged individuals (Fig. 6), Entomplasmataceae and Pseudomonaceae, are among the few families with a positive log-fold change (Table S16). However, only the increase in the Pseudomonaceae approached significance (adj. *P* = 0.09). Gram-negative and gram-positive bacteria did not differ in their response to the immune challenge (Pearson’s Chi-squared test, χ^2^ = 0.16, *P* = 0.66; Table S17).

The relative abundance of the Aerococcaceae was very similar in the immune-challenged and non-immune-challenged workers, pointing to a possible coevolution between these bacteria and their ant host. Four OTUs were assigned to the Aerococcaceae, but only one was common. An online BLAST search of this OTU sequence against the 16S bacterial/archaeal NCBI database gave the highest identity (100%) with a lactic acid bacterium, *Dolosigranulum pigrum*; so it is possibly this bacterium or a related species. Lactic acid bacteria are regularly found in insect guts, though most often from the family Lactobacillaceae [39]. One operational taxonomic unit (OTU) of the genus *Pseudomonas* (Pseudomonaceae) was present in every single gut community. The genus *Pseudomonas* is known for its metabolic diversity and ability to colonize many niches, including guts. The omnipresence of this OTU makes it also likely to be unintentionally sequenced, but this OTU was on average 23 times more abundant in our experimental samples than in our controls and thus it is unlikely to be a contaminant. A BLAST search of its 16S rRNA sequence gave hits with a variety of *Pseudomonas* species, but no other known gut inhabitants.

Bacteria belonging to the order Rhizobiales are commonly found in guts of Hymenopterans and have been hypothesized to recycle nitrogen for their host [40, 41]. Among the *T. rugatulus* gut communities, 12 rhizobial OTUs and five rhizobial families were included in our differential relative abundance tests (Bartonellaceae, Brucellaceae, Methylobacteriaceae, Methylocystaceae and Rhizobiaceae), but only the Methylocystaceae were significantly less abundant in immune-challenged workers (Table S17). The Bartonellaceae and Brucellaceae had a positive log-fold change. Only one OTU belonged to the Bartonellaceae and this OTU was not determined to the genus level. A BLAST search gave no identical hits, but a close hit was found in the NCBI Nucleotide collection. The Bartonellaceae OTU shared most 16S rRNA sequence similarity with a bacterium found in another myrmecine ant [42]. The family Brucellaceae was solely represented by an OTU assigned to the highly diverse genus *Ochrobactrum*, which encompasses mammalian pathogens, plant and soil associated bacteria, but is also regularly found as part of insect gut communities (e.g. [43, 44]). The immune challenge had no significant effect on the number of 16S rRNA gene copies, although there was a trend that immune-challenged ants harboured more rather than less bacteria (linear mixed-effects model, *N* = 24, LR = 2.87, *P* = 0.09; Fig. 7c). The three samples that had the most 16S rRNA copies were from three immune-challenged ants that had a high relative abundance of *Entomoplasma* in their gut community (Fig. 6).

### Interaction between immune gene expression and gut microbiome

Among the genes with an antimicrobial effector functionality (Table S18), seven were significantly upregulated in immune-challenged individuals and three, *abaecin*, *hymenoptaecin* and a *defensin* were associated with the Bray-Curtis distances between gut communities (Table 1). A constrained ordination analysis including only the immune challenge, and the expression (TPM) of *abaecin*, *hymenoptaecin* and *defensin*, explained 31.19% of the variation in gut communities, while *hymenoptaecin* and *defensin* remained significant (CAP, *hymenoptaecin*: F = 6.21, *P* = 0.01; *defensin*: F = 2.79, P = 0.01; Fig. 7d), immune challenge and *abaecin* were no longer significant in this model (backwards elimination, F = 1.59, *P* = 0.13 and F = 1. 25, *P* = 0.18, respectively).

**Table 1:** Results of the models testing the effect of antimicrobial gene expression on gut community composition

Effector gene	F	p
*Abaecin*	1.90	0.04*
*Defensin*	2.01	0.03*
*Defensin-2*	1.73	0.06
*Hymenoptaecin*	2.42	0.01*
*Lysozyme 2*	1.56	0.08
*Phormicin*	1.61	0.08
*Sapecin-C*	1.66	0.07
*Transferrin*	1.37	0.15

*: *p* < 0.05

## Discussion

In *Temnothorax* ants, as in many other social insects, queen removal induces worker fertility, which can cause an extension in lifespan [23]. In this study, we investigated the independent and combined effects of immune challenge and fertility induction on gut microbiome composition and fat body gene expression. Our gene expression and gut microbiome analyses showed that an immune challenge induced by an injury from a lipopolysaccharide coated needle had a drastic impact on gene expression in the fat body of workers of the ant *T. rugatulus*, while fertility induction by queen removal had a much smaller effect. Functional enrichment analyses revealed that fertile workers invest more into body maintenance, in particular in molecular repair mechanisms. Transcriptomic responses to the immune challenge depended on worker fertility, though many immune genes were activated in immune-challenged fertile and infertile workers. The immune challenge caused a dramatic drop in gut microbial diversity, while bacterial density remained constant. Microbiome changes were closely linked to changes in the expression of immune genes. Fertility induction did not affect the microbiome of ant workers and thus cannot explain their extended lifespan.

### Influence of fertility and immune challenge on fat body gene expression

Many genes altered their expression in response to an interaction between the fertility induction and immune challenge. In the absence of an immune system provocation, fertile workers focussed on degrading reactive oxygen species as indicated by the enriched function *hydrogen peroxide catabolism*. Yet, immune-challenged fertile workers activated genes of the *alpha-ketoglutarate* (*alpha-KG*) pathway. In *C. elegans*, the immune-enhancing molecule *alpha-KG* reduces the accumulation of reactive oxygen species in the mitochondria by inhibiting the ATP-synthase (sub-unit V) and reducing energy consumption [38, 45, 46]. Moreover, *alpha-KG* has been suggested to be an endogenous tumour-suppressor, to downregulate TOR and to extend lifespan in *C. elegans* [38, 46]. In insects, *alpha-KG* was not yet found to be involved in aging, but there is evidence that *alpha-KG* food supplementation enhances antioxidant capacities of female *D. melanogaster* flies [47]. In addition, in response to the immune challenge, fertile workers upregulate genes with *degradation of glutamate for alpha-KG* functionalities, while infertile workers activate stress response mechanisms, such as degradation of reactive oxygen species, but also downregulate TOR. TOR signalling has evolved in response to temporal variation in the environment by facilitating plasticity in life history traits, in particular by varying investment into somatic maintenance (*somatic maintenance polyphenism* [13];). Oxidative stress causes a downregulation of TOR signalling in *Drosophila*, which in turn increases survival and stress resistance [13, 48]. Thus, a TOR downregulation in response to an immune challenge could increase stress resistance in *T. rugatulus.*

The transcriptomic changes following fertility induction yielded few candidate genes or enriched functions. The upregulation of a *vitellogenin receptor* in fertile workers can be explained by the function of these receptors to facilitate vitellogenin up-take of eggs during maturation. Similarly, highly fecund queens of the fire ant *Solenopsis invicta* overexpress *vitellogenin receptors* in their ovaries [49]. Also in the ant *T. longispinosus* the expression of this *vitellogenin receptor* differs between queen and workers and nurses and foragers [50]. Yet in contrast to our results, *vitellogenin receptor* expression was independent of worker fertility in this congeneric species. Fertile *T. rugatulus* workers upregulate genes with the enriched function *S-adenosylmethionine biosynthetic processes*. *S-adenosylmethionin* (SAM) accumulates during aging in *Drosophila* and lifespan is extended by blocking SAM synthesis in the nematode *Caenorhabditis* or by increasing SAM catabolism in *Drosophila* flies [51, 52]. The analysis of the interaction between the immune challenge and fertility revealed that fertile workers invest more into molecular repair mechanisms such as *telomere maintenance*, *base-excision repair* and *nucleotide-excision repair* than infertile workers. Since telomere maintenance and DNA damage repair systems are strong predictors of lifespan [8, 53, 54], our findings indicate that fertile workers invest more into somatic maintenance, which could well explain their extended lifespan. Our results show weak changes in the expression of fertility genes in the fat body, indicating that this tissue plays a lesser role in the molecular regulation of fertility. In the absence of immune challenge, we found no difference in immune activity between fertile and non-fertile workers, indicating that the increased survival of fertile workers is not due to a higher basal immune activity, but rather due to an upregulation of DNA damage repair systems and possibly an alteration of the *S-adenosylmethionine* pathway.

The immune challenge resulted in a stronger transcriptomic response than the fertility indication, as the expression of more than twice as many genes was altered. Not surprising, *peptidoglycan catabolism, defence response* and *immune defence* were enriched functions in the genes upregulated in immune-challenged workers. Peptidoglycan is a main component of bacteria cell membrane and it’s catabolism represents a major step in the defence against pathogenic bacteria [55]. Unexpectedly, *activation of innate immune response*, and inosine monophosphate (IMP) synthesis was enriched among the upregulated genes of non-immune-challenged workers. Supplementation of IMP, a ribonucleotide monophosphate, positively affects survival after an immune challenge in fish [56]. Overall, our data reveal a downregulation of some aspects of the innate immune system in response to a severe immune challenge. This challenge seems to have induced oxidative stress or increased the production of radical oxygen species. We observed a similar pattern in the interaction analysis, as immune-challenged workers activated molecular repair mechanisms (*base-excision repair, nucleotide-excision repair, telomere maintenance*), which indicate a physiological cost of the immune response. Indeed, an immune reaction is costly, generating reactive oxygen species and molecular damage that have to be compensated [34, 57]. Moreover, an immune response is often accompanied by a proliferation of immune cells - haemocytes in insects [58, 59] - which can shorten telomeres [60], and thus explain the activation of telomere maintenance. All of these observations were made 24 h after the immune challenge. Thus, aspects of an ants’ immune response happening earlier or later would escape our analysis. Analysing the transcriptome of workers sampled at additional time points before and after 24 h would tell us more about the temporal aspect of the transcriptomic response following the immune challenge.

### Influence of fertility and immune challenge on the gut microbiome

Animals generally have intimate relationships with their gut microbes. Yet, the physiological, behavioural and hormonal changes that come with an increase in fecundity in worker ants, as evident also in the transcriptomic changes, did not affect the gut microbiome of *T. rugatulus* workers. Although gene expression of fertile and infertile immune-challenged ants differed markedly, this did not seem to result in differences in antimicrobial peptide production, as the microbiomes in the gut of the immune-challenged ants did not shift based on fertility. Thus, our study did not point to a role of the gut microbiome for the observed lifespan extension with worker fertility. This is surprising, as the experimental inoculation with specific gut microbes increased survival and pathogen resistance in *C. elegans* and *A. mellifera* [18, 32, 33].

However, the injury and LPS-PGN exposure caused a drastic decrease in the diversity of the gut bacterial community. In *Drosophila,* NF-κB/Relish-driven antimicrobial peptide expression is repressed to maintain a normal gut microbiome [61, 62]. Consistent with this, an immune hyperactivity affects the healthy gut microbiome in *Drosophila* and humans, resulting in disease and reduced lifespan [62–64]. Additionally, parasites seem capable of modulating antimicrobial peptide synthesis in their host to impair the growth of certain gut bacterial species that would otherwise have prevented the parasite’s establishment [65]. Our results demonstrate that a single immune challenge can cause a sharp decline in gut microbiome diversity in ants, suggesting that spill-over effects of unspecific immune flare-ups can have negative consequences for gut homeostasis.

Gut bacteria benefit their insect hosts in various ways, for example, by aiding in digestion [66, 67], protecting against pathogens [68] and increasing fecundity [69]. Diversity of the colony gut microbiome is linked to high colony productivity in another *Temnothorax* species, *T. nylanderi* [70]. Thus, a turnover of the normal gut microbiome following a provocation of the immune system somewhere else in the body could present an additional cost to immune responses in insects, next to the direct energetic costs of immune activity. To the best of our knowledge, this is the first study to demonstrate negative effects of an externally induced systemic immune response on gut bacteria in insects. If the individual survives the injury or infection that triggered the immune flare-up, the gut community may be able to re-establish by re-inoculation of bacterial strains through frequent mouth-to-mouth and mouth-to-anus contact (“trophallaxis”) with nestmates (e.g. [71] and by contact with nest material [72]). It is possible that these options to re-establish a diverse microbiome enable ants to respond so drastically to an immune challenge. The recovery time of the normal gut bacterial community after an immune flare-up should be investigated as a start to assess the costs of a loss of gut bacterial diversity after immune activity. Moreover, whether these costs depend on the bacterial species that dominate the gut after the immune flare-up is a question that should be considered, as our results revealed that two different bacterial families survive the treatment and become dominants. Interesting would also be to determine whether the bacterial strains that dominate the gut after the immune response influences the re-establishment of a normal gut community.

Testing for associations between the gut microbiome composition and the expression of several antimicrobial effector genes, allowed us to identify genes that may have killed gut bacteria after the immune challenge. Although we did not measure the expression of *hymenoptaecin, defensin* and *abaecin* in the gut tissue directly, antimicrobial peptides were both locally produced by epithelial cells and systemically in the fat body in *Drosophila*, after which they were secreted into the haemolymph [73]. Thus, the expression of *hymenoptaecin*, *abaecin* and *defensin* in the fat body may reflect systemic concentrations of their respective proteins and have a measurable effect on the gut microbiome. Insect defensins are mainly active against gram-positive rather than against gram-negative bacteria [74], while *abaecin* and *hymenoptaecin* are active against both [74, 75]. Possibly, the piercing of the cuticle itself may have kicked off an unspecific immune-flare-up.

The lack of difference in absolute bacterial abundance between the samples of immune- and non-immune-challenged ants suggests, that after the host immune response wipes out most bacterial species in the gut, unaffected bacteria species can rapidly increase in population size due to a decrease in competition. Similarly, inhibition of the intestinal homeobox gene *Caudal* in *Drosophila* led to an overexpression of antimicrobial peptide genes, resulting in the disappearance of the normal commensal bacteria population and the subsequent proliferation of a pathogen [68].

An increase in relative abundances of bacterial taxonomic groups in *T. rugatulus* after an immune flare-up may indicate special relationships between the ant host and these bacteria, not necessarily mutually beneficial ones. Bacteria from the families Aerococcaceae, Bartonellaceae, Brucellaceae, Entomplasmataceae and Pseudomonaceae did not decrease in relative abundance in the gut communities of immune-challenged ants, suggesting that these bacteria can withstand the immune defences of *T. rugatulus*. How these bacteria escaped the immune onslaught, and whether this is the result of host-symbiont coevolution, are interesting topics for future research.

## Conclusions

Our investigation of the proximate mechanisms regulating fertility and longevity supported some of our earlier predictions, as fertile workers invested more in somatic maintenance and responded differently to an immune challenge. Other expectations such as a role of the gut microbiome in the lifespan extension of fertile workers were not met. Instead, we uncovered an unpredicted dramatic reduction in gut microbial diversity caused by the immune challenge and associated with an upregulation of important immune effectors. Indeed, the injection of lipopolysaccharide challenged the immune system of ant workers so severely, that fat body gene expression responded to it much more than to the induction of fertility. The challenge apparently induced physiological stress such as oxidative stress and molecular damage, which was counter-acted by the activation of stress response and molecular repair mechanisms. The expression of additional genes was linked to an interaction between the two factors. Fertility induction only weakly altered immune investment, but fertile workers invested more into somatic repair, possibly explaining their extended lifespans. Finally, worker fertility did not alter the composition of the gut microbiome, suggesting that the latter is not involved in the lifespan extension of fertile workers. Yet the single immune challenge caused a drastic loss in gut microbiome diversity and as evidence from other *Temnothorax* ants indicate beneficial effects of a high microbiome diversity in the gut, our study revealed another potential intrinsic cost of an immune reaction, which is the loss of beneficial symbionts.

## Methods

### Ant collection and maintenance

Colonies of the ant species *Temnothorax rugatulus* were collected in the Chiricahua Mountains, Arizona USA in August–September 2015 (Table S1). A collection permit was granted by the Coronado National Forest. A total of 53 queenright monogynous colonies were used for our experiments. Colony size varied between 85 and 304 workers (mean = 143.5; SD = 68.4). After collection, colonies were kept individually in boxes (9 × 9 cm) with a humid plaster floor containing an artificial nest-site, which consisted of a Plexiglas perimeter (2 mm high) sandwiched between two microscope slides. Colonies were fed twice per week with honey and crickets and had access to a constant water supply. They were maintained in controlled climatic conditions simulating field conditions. Our study using a non-endangered insect is exempt from requiring ethical approval or permission from national and regional law.

### Queen removal and worker survival

In order to study the impact of queen removal on worker survival in our focal species, we conducted a long-term survival experiment, for which we used 45 colonies. Each colony was split in two size-matched fragments (worker number: mean = 39.5; SD = 13.8; Table S1). We labelled 2 to 6 nurses from each fragment with two coloured wire loops around the petiole (0.02 mm, Elektrisola, combination of red, green, blue or silver). Colonies were transferred to new artificial nest boxes and kept at 22 °C, 12 h/12 h light/dark. The survival of the labelled workers was recorded every 2 weeks following colony splitting for 26 weeks. The influence of the queen’s presence on survival was analysed using Cox proportional hazards model, specifically the *coxme* function of the *coxme* package implemented in *R*, Version 3.2.1. The survival model included N of days until death per individual as the response variable, queen presence (Yes, No) as explanatory variable and colony identity and fragment identity as random factors.

### Fertility induction and immune challenge

To obtain non-fertile and fertile workers for the immune challenge experiments, we used eight colonies, with a colony size varying between 34 and 93 workers (mean = 62.3; SD = 23.7). To induce worker fertility, each colony was split in half (Table S1). In each fragment four nurses, which are workers caring for the brood, were individually labelled with a coloured wire loop around the petiole (0.02 mm, Elektrisola, red or green). Young *Temnothorax* workers take over brood care and are most likely to become behaviourally dominant and start reproduction following queen removal [23, 76, 77]. Colonies were then transferred to new artificial nest boxes and kept at 25 °C, 12 h/12 h light/dark for 9 weeks. Queen removal led to a 28.6% increase in worker ovariole length (mean ± SD; queenright: 0.49 ± 0.14 mm; queenless: 0.63 ± 0.19 mm; F_1_ = 5.55, *p* = 0.027; (Fig. 1). The number of eggs in development in the ovaries did not differ between the two groups (mean ± standard error; queenright: 0.35 ± 0.13; queenless: 0.38 ± 0.14; F_1_ = 0.03, *p* = 0.86). *Temnothorax rugatulus* workers can lay male-destined, haploid eggs following queen removal and we observed worker-laid eggs in all queenless colonies during the 9 weeks following queen removal (Table S2). Due to their enlarged ovaries, we refer to workers from the queenless treatment as fertile (F; Fig. 1), and those from the queenright as infertile (NF; Fig. 1).

Nine weeks after colony separation, each nest was placed at − 20 °C for 10 min to reduce worker activity. Immediately following, the nest site was opened and each labelled worker was removed with forceps and individually isolated in a Petri dish. From each fragment, two of the four labelled workers received an immune-challenge (IC) by pricking them with a needle (diameter = 0.15 mm, length = 40 mm) dipped in a 10% lipo-polysaccharide (LPS) solution (containing lipo-polysaccharide and peptidoglycan [PGN]; provided by Sigma-Aldrich). Workers were carefully pricked through the thin cuticle between the 2nd and 3rd segment of the gaster. The non-immune-challenged workers (NIC) received the same manipulation, but the needle only touched the cuticle, not penetrating it. Thereafter, all labelled workers were returned to their colony.

### Fat body gene expression

Twenty-four hours after the immune challenge, all labelled workers (Fig. 1) were removed from their nest with soft forceps, immediately killed by decapitation and dissected on ice in 1% PBS. The fat body attached to the two segments of the gaster was crushed into 50 μL of TRIZOL and then stored at − 80 °C until RNA extraction. Dissection of each worker took less than 5 min. Ovaries were dissected and photographed with a Leica DFC425 camera at 20x magnification under a stereomicroscope. Ovary length was measured and the developing eggs were counted using Leica software (LAS version 4.5). RNA was extracted using the RNAeasy mini extraction kit (Qiagen).

Library preparation and sequencing of 100 bp paired reads was done according to standard protocol on an Illumina HiSeq 4000 (StarSeq, Mainz, Germany). We sequenced the fat body transcriptomes of eight workers from each of our four treatments, resulting in 32 sequenced samples of 45 million read pairs each (Fig. 1). The raw reads were trimmed using *Trimmomatic-v0.36* [78] and quality checked using *FastQC-v0.11.5*. Subsequently, all paired reads were assembled de novo using *Trinity* (trinityrnaseq-Trinity-v2.4.0) [79]. The final assembly contained 150.423 contigs, a mean length of 1128 bp, and a mean back mapping rate of 70.65% (details in supplement, Table S3a).

We conducted a *blastX* search against the non-redundant invertebrate protein database (state September 2017) to annotate the contigs. About 50.42% were annotated in *Blast* with 24.15% having a unique Blast hit (contig list and Blast annotation Table S3b). After translation of nucleotide sequences into amino-acid sequences with *Transdecoder-v3.0.1* (https://github.com/TransDecoder), the gene ontology (GO) and the Kyoto encyclopaedia of genes and genomes (KEGG) term annotation were performed using *InterProScan-v5.25–64.0* [80]. Read count per contig and sample was obtained by using RSEM-v1.3.0 [81], and we completed a differential gene expression analysis with the R package of Deseq2-v1.2.10 [82]. We separately tested the effects of fertility induction and immune challenge, and their interaction, while controlling for colony identity. Additionally, among the contigs that were significantly influenced by the fertility *x* immunity interaction, we compared the different subgroups (any combination of two factors): i) fertile versus infertile workers, separately and successively, within the immune-challenged workers, and within the non-immune-challenged ones; and ii) immune-challenged versus non-immune-challenged workers, separately and successively, within the fertile workers, and within the non-fertile ones. Among the list of differentially expressed genes we searched for candidate genes associated with fertility, longevity and immunity with the help of the literature, by searching for gene names and traits. Using the online tool *KEGG Mapper* (http://www.genome.jp/kegg/tool/map_pathway1.html), we conducted a pathway analysis for the upregulated contigs of interest (Table S4 and S5). In order to visualize gene expression patterns across samples categories, among the contigs, which were significantly influenced by the fertility *x* immunity interaction, we used a clustering tool (degPatterns from the ‘*DEGreport*’ R package) and plotted their relative expression per cluster (Fig. S1).

To identify biological processes associated with DEGs, we performed a functional enrichment analysis with all upregulated contigs. The enrichment analysis was done for all contigs affected by the interaction through completing separate tests for each factor level. The contigs that were upregulated in response to one treatment irrespective of the other treatment (e.g. upregulated in fertile workers compared to infertile ones irrespective of the immune challenge). We used the R package *TopGo* [83] for the enrichment analysis, and the *p*-values for each GO term were obtained with a *Fisher’s exact* test. In order to identify gene networks, a gene co-expression analysis was performed with WGCNA (Zhao et al. 2010) on the top 20,000 contigs with the highest across sample variance (details see supplement). Finally, GO terms enrichment analyses were performed on the contig list of each of the co-expression modules, as described above. All the results of the WGCNA and functional enrichment of each module are presented in the supplement (Table S6, S7 Fig. S2).

### Gut microbiome analyses

Immediately after removing the fat bodies we separated the guts without the crop from the rest of the abdomen with clean dissection tools. The guts were stored individually at − 80 °C in 10 μl 1% PBS until DNA extraction. To control for bacterial contaminants, we included three samples from the 1% PBS, in which the ants were dissected: one with only the PBS, another one in which we dipped the tools used for the dissections and one in which we dipped several intact ants to control for bacteria present on the cuticle. It was not possible to wash the ants before dissection, since this might have interfered with their gene expression. As a fourth control, we took a sample from the food of the ants. Before DNA extraction, the guts were frozen in liquid nitrogen and subsequently crushed with sterile plastic mortars. We used an industrial kit (MasterPure™, from EpiCentre, Wisconsin, USA) for DNA extraction (details in supplement)*.*

The amplification and sequencing of the 16S V4 region was performed by StarSEQ GmbH, Mainz, Germany. Amplification was done with the 515f-806rB primer pair. Overlapping, paired-end reads of 250 bp were generated with Illumina MiSeq. De-multiplexing, adapter trimming and quality filtering was done following the normal Illumina MiSeq workflow by StarSEQ. The initial data set contained 505.050 read pairs (available online see data accessibility section).

We mainly used the Uparse 9.2 pipeline [84] to determine operational taxonomic units (OTUs). Paired-end reads were merged (on average 86.2% ± 2.7 SD could be merged per sample) and subsequently filtered (maxEE cut-off set to 1). The “-fastx_uniques” step was used to de-replicate filtered reads, after which we used the “-cluster_otus” step to cluster the sequences into OTUs with a threshold of 97%. This latter step also removes chimeras. Next, the merged, unfiltered reads were mapped to the OTUs with a 97% identity cut-off with the “usearch_global” algorithm (on average 97.7% ± 1.1 SD were mapped to the OTUs per sample). Using Qiime 1.9.1 [85], we allocated taxonomic groups to the OTUs, the Greengenes database gg_13_8 as a reference. Unassigned OTUs and OTUs classified as chloroplasts or mitochondria were removed from the OTU table, but archaeal OTUs were left in. If an OTU was not at least five times more abundant in any experimental sample than in a control sample, it was discarded. Additionally, we excluded OTUs that did not make up at least 0.1% of the community in at least one sample to further eliminate contaminations. The resulting OTU table contained 378 taxonomic units.

Total bacterial 16S copy numbers per ng DNA were quantified by quantitative realtime PCR (qrt-PCR) with universal 16S primers (details see supplement). The total DNA concentration of each sample in ng/μl was determined with a Qubit Fluorimeter (Thermofisher). For each replicate, we calculated the amount of bacterial 16S copies per ng of DNA in a sample. The means of the replicates were log transformed and subsequently compared between non-immune-challenged and immune-challenged individuals using linear mixed-effects (LME) models from the R package nlme, including colony as random effect.

The OTU table was rarefied to a depth of 1500 reads. Using the rarefied table, we calculated the pairwise Bray-Curtis dissimilarities between the samples in Qiime. Samples were clustered based on their Bray-Curtis dissimilarities (average linkage method, 1000 bootstraps) with the pvclust package in R [86]. Clusters assigned AU values higher than 98.5 are highly supported. The OTUs were binned according to microbial family and the relative abundance of each microbial family was calculated for each sample. This data was used to generate a stacked bar plot, which was combined with the pvclust tree plotted with the R package ggtree [87], to visualize the differences in community composition between samples.

To compare the bacterial diversity between the treatments for each sample we calculated the Shannon’s diversity index with the alpha_diversity.py script of Qiime using the rarefied table. Additionally, we computed Simpson’s diversity index, which gives more weight to common species than Shannon’s diversity, with the *diversity* function of the R package *vegan*. We tested for an effect of fertility, immune challenge and their interaction on both measures of diversity with LME models: The fragment and the colony of the ant were included as a nested random effect term. Simpson’s diversity index was arcsine square root-transformed to improve normality. Model residuals were visually checked for deviation from normality.

To identify taxonomic groups that differed in relative abundance between treatments we binned the OTUs into their respective microbial families. To restrict the analysis to the more important taxa, the microbial families that were present in less than 15% of the samples were removed from the OTU table. For the differential abundance testing we performed permutation tests as implemented in the *fitLogNormal* function of the metagenomeSeq R package [88], using 100 permutations. The *p*-values were adjusted with the “FDR” method. Taxonomic features with adjusted p-values smaller than 0.05 were regarded as significantly different in relative abundance between the treatments.

We were interested whether variation in *T. rugatulus* gut communities could be explained by the expression of effector genes involved in antimicrobial immunity. Therefore we looked for the presence of such genes among the contigs and checked whether these contigs were significantly differentially higher expressed in immune-challenged compared to non-immune-challenged individuals. If multiple contigs were annotated with the same effector gene name, we chose the contig with the highest average expression (TPM). With the resulting set of contigs we completed a constrained ordination analysis with the *capscale* function of the R package *vegan* (distance-based redundancy analysis, Bray-Curtis) [89]. To test for an association between the expressions of the antimicrobial effector genes on the gut communities, we compared two models with an ANOVA for each contig separately: one model with only fixed factors and one model with next to the fixed factors the expression of the contig (TPM).

## Supplementary Information

**Additional file 1.** Additional methodological details, Figures S1 and S2 and Table S2. Description of additional supplementary tables (Tables S1, S3-S18) are also provided.

## Data Availability

All sequence data for this study were archived at NCBI’s Short Read Archive (SRA) with links to BioProject accession numbers PRJNA509332 and PRJNA513968 (https://www.ncbi.nlm.nih.gov/bioproject/).

## Abbreviations

*ROS*: *Reactive oxygen species*
IIS: *Insulin/insulin-like* growth factor
TOR: Target of rapamycin
OTU: Operational taxonomic unit
TPM: Highest average expression
CAP: Capscale
Alpha-KG: *Alpha-ketoglutarate* pathway
*SAM*: *S-adenosylmethionin*
IMP: *Inosine monophosphate*
KEGG: Kyoto encyclopedia of genes and genomes
GO: Gene ontology
LME: Linear mixed-effects
WGCNA: Weighted gene co-expression analysis
FDR: False discovery rate
IC: Immune-challenged workers
NIC: Non-immune challenged workers
F: Fertile workers
NF: Infertile workers
