# Supplementary material for: Immune challenge reduces gut microbial diversity and triggers fertility-dependent gene expression changes in a social insect
Source: BMC Genomics. 2020 Nov 23;21:816. doi: 10.1186/s12864-020-07191-9 (PMC7682046; doi:10.1186/s12864-020-07191-9)
Supplement: Supplementary file 1 — Additional file 1. Additional methodological details, Figures S1 and S2 and Table S2. Description of additional supplementary tables (Tables S1, S3-S18) are also provided. [file 12864_2020_7191_MOESM1_ESM.zip › Supplemental Information BMC Genomics.pdf]

Supplemental Information

Immune challenge reduces gut microbial diversity and triggers fertility-dependent gene expression changes in a social insect

Matteo Antoine Negroni, Francisca H.I.D. Segers, Fanny Vogelweith, Susanne Foitzik

Table of Contents:

1. **Details for the *de novo* transcriptome assembly**
2. **Details for Weighted Gene Co-expression Network Analysis (WGCNA)**
3. **Details for the DNA extraction from gut samples**
4. **Details for total bacterial 16S DNA quantification**
5. **Figure S1**
6. **Figure S2**
7. **Table S1**
8. **Table S2**
9. **Table S3**
10. **Table S4**
11. **Table S5**
12. **Table S6**
13. **Table S7**
14. **Table S8**
15. **Table S9**
16. **Table S10**
17. **Table S11**
18. **Table S12**
19. **Table S13**
20. **Table S14**
21. **Table S15**
22. **Table S16**
23. **Table S17**
24. **Table S18**

Details for the *de novo* transcriptome assembly

Library preparation and sequencing of 100bp paired reads was done according to standard protocol on an Illumina HiSeq 4000 (StarSeq, Mainz, Germany). In total, we sequenced the fat body transcriptomes of eight workers of our four treatments, resulting in 32 sequenced samples of 45 million read pairs each (Fig. 1). The raw reads were trimmed using *Trimmomatic-v0.36* (Bolger et al. 2014), and the quality checked using *FastQC-v0.11.5*. Subsequently, all paired reads were assembled *de novo* using *Trinity* (trinityrnaseq-Trinity-v2.4.0) (Haas et al. 2013). Due to the large amount of data (± 45 million read pairs per sample), we were unable to assemble all paired reads at once. Thus, we generated two assemblies, each with half of our samples, ensuring an equal amount of each treatment in each set of the samples. We subsequently merged the two assemblies by first removing the identical contigs with the program *CD-HIT-EST* (Li and Godzik 2006) and then merging contigs with CAP3 (Huang and Madan 1999). The initial two assemblies encompassed 156.371 and 151.820 contigs, while the merged assembly included 150.423 contigs (mean length of 1128 bp, back mapping rate of 70.65 % on average; Table S2a).

Details for Weighted Gene Co-expression Network Analysis (WGCNA)

In order to identify gene networks, a gene co-expression analysis was performed with WGCNA (Zhao et al. 2010) on the top 20,000 contigs with the highest across sample variance. We first constructed a gene network with all samples, with the minimal number of contigs per cluster set to 150 and a soft thresholding power set to 9 (dissimilarity threshold of 0.15), and then extracted the eigengene values for each sample and each module. As the sample clustering analysis revealed no obvious outlier, no sample was discarded for the WGCNA. In order to test module trait association according to fertility and immune challenge, we successively ran several models with both the two-level factor "fertility" in interaction with the two level factor "immune challenge" as explanatory variables, and the eigengene value of a given module as the response variable. In order to ensure normality of the residuals, we conducted the analyses across 10,000 permutations of the raw eigengene value data. The resulting p-values were adjusted for false discovery rate (FDR). Finally, GO terms enrichment analyses were performed on the contig list of each of the co-expression modules, as described above. All the results of the WGCNA and functional enrichment of each module can be found in the supplement (module-trait association results: Table S17, Fig. S3, module enrichments: Table S18).

Details for the DNA extraction from gut samples

For DNA extraction, we used an industrial kit (MasterPure™, from EpiCentre, Wisconsin, USA). We added an extra lysozyme step to the manufacturer's protocol to ensure the lysis of gram-positive bacterial cell walls: we added 2µl of ready-to-lyse lysozyme (250U/µl TES buffer) to each sample. Subsequently they were incubated for 30 min. at 37°C. The resulting DNA samples were stored at -80°C until sequencing.

Details for total bacterial 16S DNA quantification

We conducted qrt-PCR on a micPCR (Bio Molecular Systems) using a Biozym Blue S'Green qPCR Kit and universal 16S primers (Forward: 5'-ACTCCTACGGGAGGCAGCAGT-3'; Reverse: 5'-TATTACCGCGGCTGCTGGC-3'). Reactions took place in a volume of 20µl containing 6.4µl of ddH2O - 0.8µl of each primer (10µM), 10µl of Sybr mix and 2µl of the extracted DNA per sample. All runs consisted of an initial 2 min. initiation step at 95°C, 40 cycles of 95°C for 10 s, 64°C for 15 s (initially 6 touchdown steps starting from 70°C) and 72°C for 10 s. Each run was concluded with a dissociation curve analysis. All samples were replicated three times in the same run. Each run also included two to three negative controls with 2µl of ddH_2O added instead of DNA. We excluded replicates with double peaked melting curves, which had Cq values that exceeded 0.5 from the other two replicates. The DNA concentration of each sample was deducted from a standard curve made with a tenfold dilution series (10^4 to 10^{10}) of a mixture of 16S DNA copies of the bacterial associates of a firebug (*Pyrrhocoris apterus*). Due to a depletion of some samples for the Illumina MiSeq sequencing and the exclusion of samples based on double peaked melting curves, we were left with 11 samples from non-immune-challenged individuals and 13 samples of immune-challenged ants.

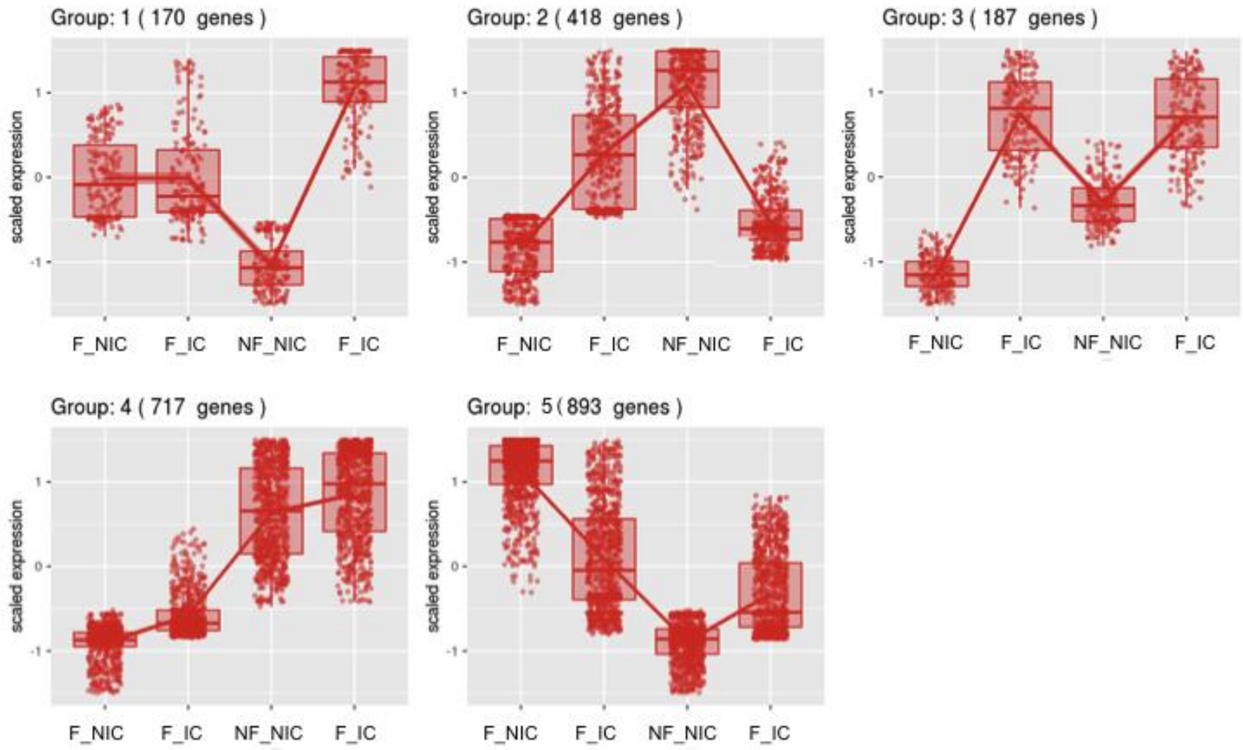

Fig S1. Plots of relative expression of the gene, per cluster (group) according to their expression pattern, based on the list of contigs (N = 2,487 contigs) interacting in their expression in the fertility x immune-challenge interaction (F: Fertile; NF: non-fertile; IC: immune challenged; NIC: non-immune challenged).

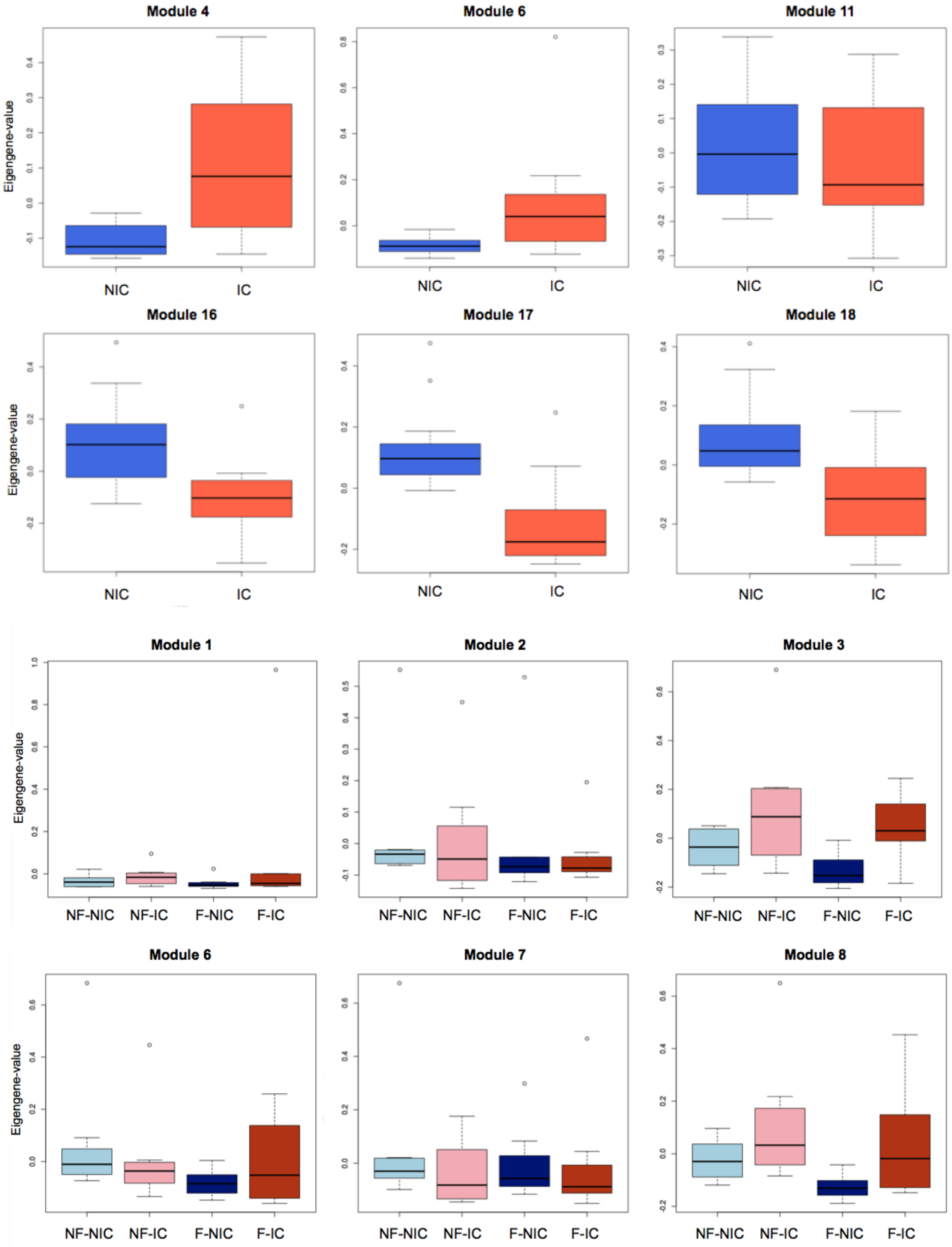
Module 4
Eigengene-value
NIC
IC
Module 6
NIC
IC
Module 11
NIC
IC
Module 16
Eigengene-value
NIC
IC
Module 17
NIC
IC
Module 18
NIC
IC
Module 1
Eigengene-value
NF-NIC
NF-IC
F-NIC
F-IC
Module 2
NF-NIC
NF-IC
F-NIC
F-IC
Module 3
NF-NIC
NF-IC
F-NIC
F-IC
Module 6
Eigengene-value
NF-NIC
NF-IC
F-NIC
F-IC
Module 7
NF-NIC
NF-IC
F-NIC
F-IC
Module 8
NF-NIC
NF-IC
F-NIC
F-IC

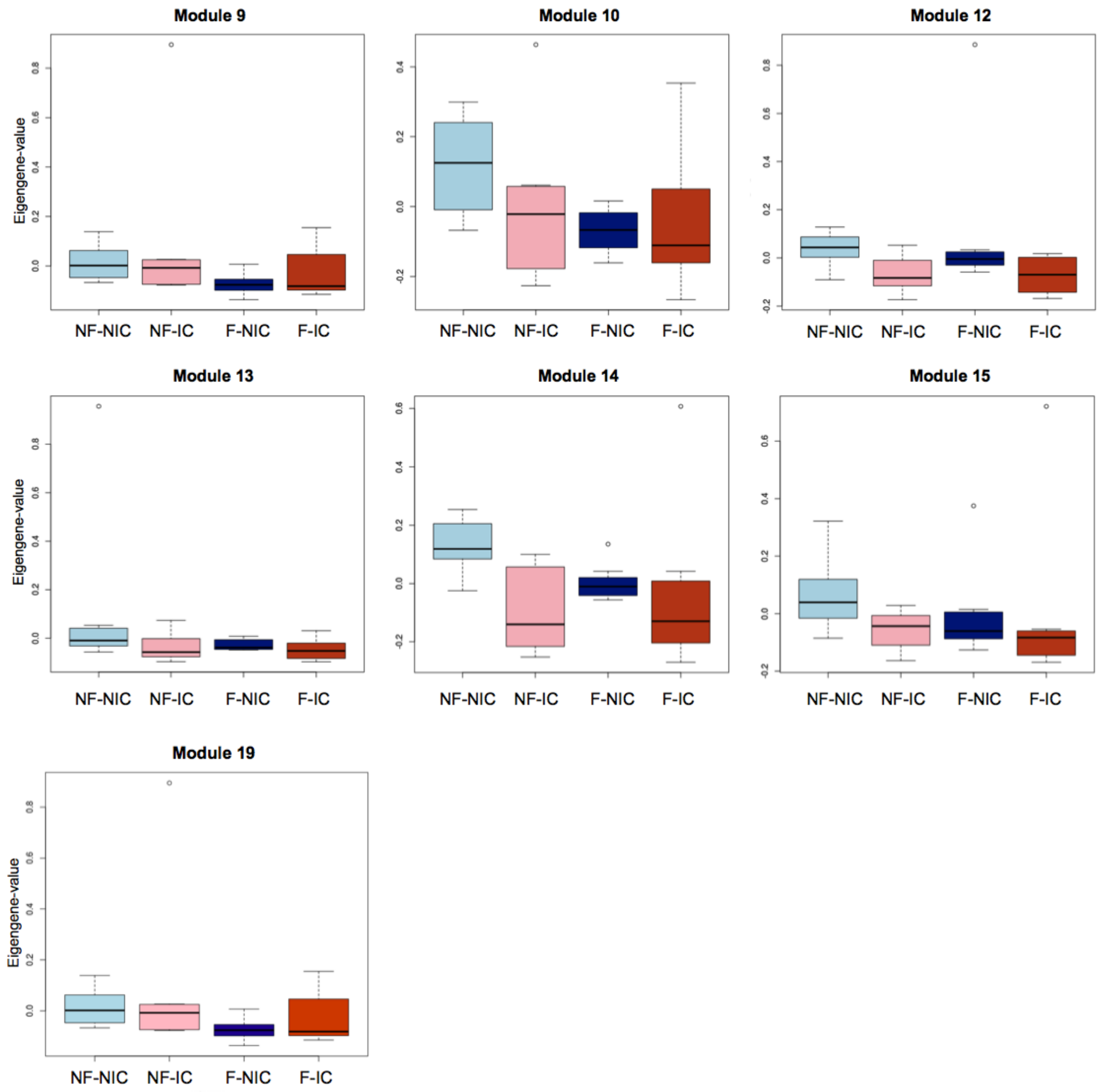

Fig S2. Module-traits association testing for the influence of fertility (F: fertile, NF: non-fertile workers) in interaction with immune challenge (IC: immune-challenged, NIC: non-immune-challenged) on the eigengene value, for each module with on top the ones that were significantly influenced by at least one factor (*immune-challenge / fertility / immune-challenge x fertility* interaction), implemented with colony identity as random factor. Module 19 contains all genes that could not be grouped into a co-expression module.

Table S1: The coordinates of the locations where the *T. rugatulus* colonies were collected and the number of workers in each nest after colonies were split in a queen right (QR) and queen less (QL) fragment.

Table S2: Number of eggs produced per nest and per week, for queenright (QR) and queenless (QL) each fragment. The experiment was performed in two cohorts separated by 24h.

Nest	Colony identity	Cohort	Week2	Week4	Week6	Week8
QR	A	II	2	6	4	3
QL	A	II	12	35	25	20
QR	B	I	19	29	18	10
QL	B	I	0	0	1	1
QR	C	II	10	13	23	42
QL	C	II	0	0	0	1
QR	D	II	8	9	11	25
QL	D	II	0	7	3	1
QR	E	II	10	12	9	10
QL	E	II	0	0	1	0
QR	F	II	2	5	6	8
QL	F	II	0	0	0	1
QR	G	II	33	41	32	27
QL	G	II	12	31	21	17
QR	H	I	20	17	14	10
QL	H	I	3	14	10	12

Table S3: Back mapping rate of each paired read per sample a) and BlastX annotation b) Sample identity is a combination of colony identity (A, B, C, D, E, F, G, H), the fertility status (fertile: F or non-fertile: NF) and the treatment with an immune challenge or not (non-immune challenged: NIC or immune challenged: IC).

Table S4: Results from the pathway analysis based, among the contigs showing an interaction in their expression, on a) the list of contigs upregulated in non-fertile workers compared to fertile ones, specifically within non-immune challenged workers; b) the list of contigs upregulated in non-fertile workers compared to fertile ones, both within the non-immune challenged workers and the immune challenge ones; c) the list of contigs upregulated in non-fertile workers compared to fertile ones, specifically within non-immune challenged workers; d) the list of contigs upregulated in fertile workers compared to non-fertile ones, specifically within immune challenged workers; e) the list of contigs upregulated in fertile workers compared to non-fertile ones, both within the non-immune challenged workers and the immune challenged ones; f) the list of contigs upregulated in fertile workers compared to non-fertile ones, specifically within immune challenged workers; g) the list of contigs upregulated in non-immune challenged workers compared to immune challenged ones, specifically within non-fertile workers; h) the list of contigs upregulated in non-immune challenged workers compared to immune challenged ones, both within the non-fertile workers and the fertile ones; i) the list of contigs upregulated in non-immune challenged workers compared to immune challenged ones, specifically within non-fertile workers; j) the list of contigs upregulated in immune challenged workers compared to non-immune challenged ones, specifically within fertile workers; k) the list of contigs upregulated in immune challenged workers compared to non-immune challenged ones, both within the non-fertile workers and the fertile ones; l) the list of contigs upregulated in immune challenged workers compared to non-immune challenged ones, specifically within fertile workers.

Table S5: Results from the pathway analysis based on: the list of upregulated contigs in a) non-fertile workers, b) fertile ones from the comparison between fertile and non-fertile worker independent from the immune challenge, and the list of upregulated contigs in c) non-immune-challenged workers, d) immune challenged ones from the comparison between immune-challenged and non-immune-challenged worker independently from fertility.

Table S6: Effect of fertility in interaction with the immune challenge on each module eigengene value. In order to ensure normality of the residuals, we conducted the analyses across 10.000 permutations of the raw eigengene value data. The resulting p-values were adjusted for false discovery rate.

Table S7: Results from the functional enrichment analysis for each co-expression module (1-19).

Table S8: List of contigs whose expression depends on the interaction between immune challenge and fertility induction (FDR < 0.05), with their BlastX annotation.

Table S9: Results of the differential gene expression analysis, testing the effect of fertility separately within immune-challenged workers and within non-immune-challenged ones, with a) the list of upregulated contigs in non-fertile workers compared to fertile ones (FDR < 0.05), within non-immune-challenged workers; b) the list of upregulated contigs in fertile workers compared to non-fertile ones (FDR < 0.05), within non-immune challenged workers; and c) the list of upregulated contigs in non-fertile workers compared to fertile ones (FDR < 0.05), within the immune-challenged workers; d) the list of upregulated contigs in fertile workers compared to non-fertile ones (FDR < 0.05), within the immune-challenged workers.

Table S10: Results of the differential gene expression analysis, testing the effect of the immune challenge separately within non-fertile workers and within non-fertile ones, with a) the list of upregulated contigs in non-immune-challenged workers compared to immune-challenged ones (FDR < 0.05), within the non-fertile workers; b) the list of upregulated contigs in non-immune-challenged workers compared to immune-challenged ones (FDR < 0.05), within the non-fertile workers; and c) the list of upregulated contigs in non-immune-challenged workers compared to immune-challenged ones (FDR < 0.05), within the fertile workers; d) the list of upregulated contigs in immune-challenged workers compared to non-immune-challenged ones (FDR < 0.05), within the fertile workers.

Table S11: Results from the functional enrichment analysis, among the contigs showing an interaction in their expression, based on a) the list of contigs upregulated in non-fertile workers compared to fertile ones, specifically within non-immune-challenged workers; b) the list of contigs upregulated in non-fertile workers compared to fertile ones, both within the non-immune-challenged workers and the immune-challenged ones; c) the list of contigs upregulated in non-fertile workers compared to fertile ones, specifically within non-immune-challenged workers; d) the list of contigs upregulated in fertile workers compared to non-fertile ones, specifically within immune-challenged workers; e) the list of contigs upregulated in fertile workers compared to non-fertile ones, both within the non-immune-challenged workers and the immune-challenged ones; f) the list of contigs upregulated in fertile workers compared to non-fertile ones, specifically within immune-challenged workers.

Table S12: Results from the functional enrichment analysis based, among the contigs showing an interaction in their expression, on a) the list of contigs upregulated in non-immune-challenged workers compared to immune-challenged ones, specifically within non-fertile workers; b) the list of contigs upregulated in non-immune-challenged workers compared to immune-challenged ones, both within the non-fertile workers and the fertile ones; c) the list of contigs upregulated in non-immune-challenged workers compared to immune-challenged ones, specifically within non-fertile workers; d) the list of contigs upregulated in immune-challenged workers compared to non-immune-challenged ones, specifically within fertile workers; e) the list of contigs upregulated in immune-challenged workers compared to non-immune-challenged ones, both within the non-fertile workers and the fertile ones; f) the list of contigs upregulated in immune-challenged workers compared to non-immune-challenged ones, specifically within fertile workers.

Table S13: Results from the differential expression analysis of the comparison between fertile and non-fertile workers independent of the immune challenge treatment; with a) the list of upregulated contigs in fertile workers compared to non-fertile ones (FDR < 0.05); and b) the list of upregulated contigs in non-fertile workers compared to fertile ones (FDR < 0.05); with their BLASTx annotation.

Table S14: Results from the functional enrichment analysis based on: the list of upregulated contigs in a) non-fertile workers b) fertile ones from the comparison between fertile and non-fertile worker independent from the immune challenge; and the list of upregulated contigs in a) non-immune-challenged workers, b) immune-challenged ones from the comparison between immune-challenged and non-immune-challenged worker independent from fertility.

Table S15: Results from the differential expression analysis of the comparison between immune-challenged and non-immune-challenged workers independent of fertility; with a) the list of upregulated contigs in immune-challenged workers compared to non-immune-challenged ones (FDR < 0.05); and b) the list of upregulated contigs in non-immune-challenged workers compared to immune-challenged ones (FDR < 0.05); with their BLASTx annotation.

Table S16: Results from the permutation tests - the abundances of the families were compared between the gut communities from fertile and non-fertile individuals. The log fold changes are relative to the samples originating from fertile ants. The P-values were adjusted with the "FDR" method.

Table S17: Results from the permutation tests - the abundances of the families were compared between immune challenged and non-immune-challenged individuals. The log fold changes are relative to the samples originating from non-immune-challenged ants. Families that differ significantly in abundance between the treatments are in italics. The P-values were adjusted with the "FDR" method. The last column lists the number of non-immune-challenged individuals that hosted the respective microbial family.

Table S18: List of all contigs annotated as antimicrobial effector genes. Some effector genes had annotated isoforms. The last column indicates whether a contig was found to be significantly differentially expressed between immune challenged and non-immune-challenged individuals.

References

Huang X, Madan A. 1999. CAP3: A DNA sequence assembly program. *Genome Res*. 9:868-877.

Li W, Godzik A. 2006. Cd-hit: a fast program for clustering and comparing large sets of protein or nucleotide sequences. *Bioinformatics*. 22:1658-1659.
